# Impact of neoadjuvant chemotherapy on the safety and long-term outcomes of patients undergoing immediate breast reconstruction after mastectomy

**DOI:** 10.1007/s12282-024-01570-w

**Published:** 2024-04-04

**Authors:** Hiroko Nogi, Akiko Ogiya, Makoto Ishitobi, Chikako Yamauchi, Hiroki Mori, Ayaka Shimo, Kazutaka Narui, Naomi Nagura, Hirohito Seki, Shinsuke Sasada, Teruhisa Sakurai, Tadahiko Shien, The Collaborative Study Group of Scientific Research of The Japanese Breast Cancer Society

**Affiliations:** 1 Department of Breast and Endocrine Surgery, The Jikei University School of Medicine, 3-25-8, Nishi-Shinbashi, Minto-Ku, Tokyo, 105-8461 Japan; 2 Department of Breast Surgical Oncology, Cancer Institute Hospital, Japanese Foundation for Cancer Research, Tokyo, Japan; 3 Department of Breast Surgery, Japanese Red Cross Medical Center, Tokyo, Japan; 4 Department of Breast Surgery, Mie University School of Medicine, Mie, Japan; 5 Department of Radiation Oncology, Shiga General Hospital, Shiga, Japan; 6 Department of Plastic and Reconstructive Surgery, Tokyo Medical and Dental University, Tokyo, Japan; 7 Department of Breast and Endocrine Surgery, St. Marianna University School of Medicine, Kanagawa, Japan; 8 Department of Breast and Thyroid Surgery, Medical Center, Yokohama City University, Kanagawa, Japan; 9 Department of Breast Surgical Oncology, St Luke’s International Hospital, Tokyo, Japan; 10 Department of Breast Surgery, Kyorin University School of Medicine, Tokyo, Japan; 11 Department of Surgical Oncology, Research Institute for Radiation Biology and Medicine, Hiroshima University, Hiroshima, Japan; 12 Sakurai Breast Clinic, Wakayama, Japan; 13 Department of Breast and Endocrine Surgery, Okayama University Hospital, Okayama, Japan

**Keywords:** Breast cancer, Neoadjuvant chemotherapy, Immediate breast reconstruction, Postoperative complications, Locoregional recurrence

## Abstract

**Background:**

In breast cancer patients receiving neoadjuvant chemotherapy (NAC), immediate breast reconstruction (IBR) as a breast cancer treatment option remains controversial. We assessed the impact of NAC on surgical and oncological outcomes of patients undergoing IBR.

**Methods:**

This was a retrospective multicenter study of 4726 breast cancer cases undergoing IBR. The rate of postoperative complications and survival data were compared between IBR patients who received NAC and those who did not receive NAC. Propensity score matching analysis was performed to mitigate selection bias for survival.

**Results:**

Of the total 4726 cases, 473 (10.0%) received NAC. Out of the cases with NAC, 96 (20.3%) experienced postoperative complications, while 744 cases (17.5%) without NAC had postoperative complications. NAC did not significant increase the risk of complications after IBR (Odds ratio, 0.96; 95%CI 0.74–1.25). At the median follow-up time of 76.5 months, 36 patients in the NAC group and 147 patients in the control group developed local recurrences. The 5-year local recurrence-free survival rate was 93.1% in the NAC group and 97.1% in the control group. (*P* < 0.001). After matching, there was no significant difference between the two groups.

**Conclusion:**

IBR after NAC is a safe procedure with an acceptable postoperative complication profile.

## Introduction

Breast cancer is the most prevalent malignancy affecting women worldwide, including Japanese women [1, 2]. In recent years, neoadjuvant chemotherapy (NAC) has gained widespread use in early-stage breast cancer patients. Initially, NAC was employed to convert inoperable locally advanced breast cancers into operable tumors and to downstage such tumors allowing breast-conserving surgery [3, 4]. However, NAC is now recognized for its role in improving outcomes through residual disease-guided approach [5]. Patients with residual tumors after NAC have various indications for adjuvant therapies such as trastuzumab emtansine for human epidermal growth factor receptor-2 (HER2) -positive tumors, capecitabine for triple-negative tumors, Poly (ADP-ribose) polymerase inhibitors for those with BRCA pathogenic variants, cyclin-dependent kinase 4/6 inhibitors, and S-1 for hormone receptor-positive, HER2-negative tumors [6–10].

As another comprehensive component of breast cancer care, immediate breast reconstruction (IBR) after mastectomy increased with the establishment of IBR procedures. IBR helps restore body image and has a positive impact on psychological well-being and quality of life for patients [11–13]. Moreover, the use of nipple-sparing mastectomy (NSM) and skin-sparing mastectomy (SSM) procedures have increased, leading to improved aesthetic outcomes. Therefore, IBR with NSM/SSM techniques has emerged as an important surgical option after NAC [14]. Despite the advantages of IBR, there is a persistent concern among physicians that IBR after NAC may delay adjuvant therapy due to postoperative complications and thereby increase the risk of locoregional recurrence [15–18]. Additionally, post-mastectomy radiation therapy (PMRT) is reportedly associated with a higher incidence of adverse cosmetic outcomes in patients undergoing IBR [19, 20].

Several studies have shown that IBR after NAC does not significantly increase complications [21–26], and previous studies have reported the prognosis of breast cancer patients with IBR after NAC is comparable to those with mastectomy alone after NAC [14, 27, 28], but it is still under debate. Therefore, we conducted a multicenter, retrospective, observational study in collaboration with the Study Group of Scientific Research of the Japan Breast Cancer Society. We aimed to investigate the potential impact of NAC on postoperative complications and locoregional recurrence in breast cancer patients undergoing IBR.

## Patients and methods

We conducted a retrospective review of the medical records of 4726 consecutive breast cancer patients who underwent IBR between January 2008 and December 2016. The data were collected from 12 institutes and included various clinicopathological factors such as menopause status, body mass index (BMI), smoking status, pathological tumor size, nodal status, estrogen receptor (ER) expression, progesterone receptor (PgR) expression, and HER2 expression, the presence of lymphovascular invasion, and surgical margin status. Information on treatment modalities, including surgical technique, reconstruction type, axillary surgery, adjuvant therapy, NAC, and PMRT was also collected. Furthermore, data on wound complications, including infection, hemorrhage, seroma, dehiscence, flap necrosis, seroma, marginal necrosis, nipple necrosis respectively after initial surgery, capsular contracture, malrotation, rupture and loss of prosthesis and loss of total flap as well as oncologic outcomes including local and regional recurrence, were examined.

This study was conducted in accordance with the Helsinki Declaration of 1975, as revised in 1983, and was approved by the Ethics Committee of each participating institute. Due to the retrospective nature of the study, the requirement for informed consent from the patients was waived.

ER and PgR expressions were assessed using immunohistochemistry, and tumors in which 1% or more of tumor cells stained positive were classified as ER or PgR positive. HER2 status was considered positive if the immunohistochemistry result was 3 + or confirmed by fluorescence in situ hybridization with an amplification ratio of ≥ 2.0. The evaluation of ER, PgR, and HER2 statuses was performed by each participating institution. BMI was classified as normal (BMI 18–24) or overweight (BMI ≥ 25). We divided postoperative complications according to the Clavien-Dindo classification. Local recurrence was defined as the presence of any breast cancer in the ipsilateral breast, skin, subcutaneous tissue, pectoralis muscle, or thoracic wall. Regional recurrence was defined as metastases involving the axillary, supraclavicular, or internal mammary lymph nodes. PMRT was administered in some cases where the surgical margins were exposed, or lymph nodes were positive for metastasis, but criteria for administering radiotherapy varied among institutions.

### Statistical analysis

All data analyses were conducted using Stata statistical software (Stata SE 13.1; Stata Corp LP, College Station, TX, USA). The patients were divided into two groups: the NAC group consisted of patients who received NAC, the control group of patients who did not. The associations between clinicopathological factors and patient groups were statistically compared using Fisher's exact test or the Mann–Whitney *U* test. Similarly, Fisher's exact test was used to analyze the associations between postoperative complications and patient groups. Additionally, the odds ratios (ORs) were estimated using logistic regression analyses to assess the effects of variables such as BMI, smoking status, NAC, type of mastectomy or reconstruction, and PMRT on postoperative wound complications. Local recurrence-free survival (LRFS) and regional recurrence-free survival (RRFS) was calculated from the time of surgery until the detection of recurrence or the final contact with the patient. Survival curves and the cumulative incidence of events were generated using the Kaplan–Meier method. The differences in Kaplan–Meier curves were assessed using the log-rank test. The hazard ratios (HRs) and 95% confidence intervals (CIs) were estimated using the Cox regression model to identify potential prognostic indicators. To mitigate selection bias in the administration of NAC, we performed propensity score matching analysis with a logit-based model using the psmatch2 STATA function. The covariates for matching were pT, pN, hormone receptor and HER2 status as factors significantly different between the NAC and control groups. All statistical tests were two-sided, and a *P* value of less than 0.05 was considered to indicate a statistically significant association.

## Results

In total 4,726 cases who underwent IBR were included in this study. Among them, 473 cases (10.0%) received NAC. The median age of patients in the NAC group was 45 years (range 24–77), while that of the control group was 46 years (range 20–83), (*P* < 0.001). Table 1 lists the characteristics of patients, tumors, and treatments for each group. There were significantly more cases with bilateral breast cancer in the control group (695 cases; 16.3%) than in the NAC group (60 cases; 12.7%) (*P* = 0.04). There were no significant differences between the two groups in terms of menopausal status, smoking habit, and BMI. NSM was performed significantly more frequently in the NAC group (190 cases; 40.2%), than in the control group (1241 cases; 29.2%) (*P* < 0.001). Reconstruction with autologous (including free-flap or pedicle-flap) or direct silicone breast implant (SBI) was performed significantly more frequently in the NAC group (96 cases; 20.3%, 51 case; 10.8%, respectively) than in the control group (427 cases; 10.0%, 115 cases; 2.7%, respectively) (*P* < 0.001). Reconstruction with tissue expander (TE) followed by SBI was performed significantly more frequently in the control group (3711 cases; 87.3%) than in the NAC group (326 cases; 68.9%) (*P* < 0.001). Axillary dissection was also performed significantly more frequently in the NAC group (260 cases; 55.0%) than in the control group (555 cases; 13.1%) (*P* < 0.001). Additionally, PMRT was performed significantly more frequently in the NAC group (115 cases; 24.3%) than in the control group (244 cases; 5.7%) (*P* < 0.001). The NAC group had significantly larger pathological tumor sizes than the control group (*P* < 0.001). Furthermore, the NAC group had higher proportions of tumors that were ER-negative (26.8%), PgR-negative (46.0%), HER2-positive (29.2%), and/or showed lymphovascular infiltration (30.0%), as compared to the control group (ER-negative, 11.4%; PgR-negative, 17.8%; HER2-positive, 12.6%; lymphovascular infiltration-positive, 22.5%). There was no significant difference in the frequency of tumor cells exposure at the surgical margin between the two groups, with 4.7% of the NAC group and 5.9% of the control group having positive surgical margins.

**Table 1:** Patient and tumor characteristics

	Total		Control		NAC		*P* value
	N = 4726		N = 4253		N = 473		
	N	%	N	%	N	%	
Age; median (range)	45	(20–83)	46	(20–83)	45	(24–77)	< 0.001
Bilateral							0.04
Yes	755	16.0	695	16.3	60	12.7	
No	3971	84.0	3558	83.7	413	87.3	
Menopause							0.62
Pre	3614	76.5	3259	76.6	355	75.1	
Post	1051	22.2	938	22.1	113	23.9	
Unknown	61	1.3	56	1.3	5	1.1	
Smoking habit							0.92
Yes	996	21.1	897	21.1	99	20.9	
No	3486	73.8	3138	73.8	348	73.6	
Unknown	244	5.1	218	5.1	26	5.5	
Body mass index							0.14
≥ 25	688	14.6	605	14.2	83	17.6	
< 25	3996	84.6	3609	84.9	387	81.8	
Unknown	42	0.9	39	0.9	3	0.6	
Breast surgery type							< 0.001
Total mastectomy	1702	36.0	1564	36.8	138	29.2	
SSM	1593	33.7	1448	34.1	145	30.7	
NSM	1431	30.3	1241	29.2	190	40.2	
Reconstruction							< 0.001
TE-SBI	4037	85.4	3711	87.3	326	68.9	
Direct SBI	166	3.5	115	2.7	51	10.8	
Autologous (Free, Pedicled)	523	11.1	427	10.0	96	20.3	
Axillary surgery							< 0.001
None	52	1.1	47	1.1	5	1.1	
SNB	3859	81.7	3651	85.9	208	44.0	
Ax	815	17.3	555	13.1	260	55.0	
PMRT							< 0.001
Yes	359	7.6	244	5.7	115	24.3	
No	4357	92.2	4001	94.1	356	75.3	
Unknown	10	0.2	8	0.2	2	0.4	
Adjuvant chemotherapy							< 0.001
Yes	1065	22.5	1014	23.8	51	10.8	
No	3643	77.1	3225	75.8	418	88.4	
Unknown	18	0.4	14	0.3	4	0.8	
pT							0.014
T0	1371	29.0	1281	30.1	90	19.0	
T1	1271	26.9	1196	28.1	75	15.9	
T2	959	20.3	885	20.8	74	15.6	
T3	850	18.0	724	17.0	126	26.6	
T4	199	4.2	135	3.2	64	13.5	
Unknown	76	1.6	32	0.8	44	9.3	
pN							< 0.001
Negative	3703	78.4	3419	80.4	284	60.0	
1–3	760	16.1	644	15.1	116	24.5	
≥ 4	211	4.5	143	3.4	68	14.4	
Unknown	52	1.1	47	1.1	5	1.1	
Histology							0.09
Ductal	4332	91.7	3893	91.6	439	92.8	
Others	393	8.3	359	8.4	34	7.2	
Estrogen receptor							< 0.001
Positive	3985	84.3	3659	86.0	326	68.9	
Negative	612	13.0	485	11.4	127	26.8	
Unknown	129	2.7	109	2.6	20	4.2	
Progesterone receptor							< 0.001
Positive	3618	76.6	3383	79.5	235	49.7	
Negative	976	20.7	758	17.8	218	46.0	
Unknown	132	2.8	112	2.6	20	4.2	
HER2							< 0.001
Positive	673	14.2	535	12.6	138	29.2	
Negative	3495	73.9	3192	75.0	303	64.1	
Unknown	558	11.8	526	12.4	32	6.8	
Lymphovascular invasion							< 0.001
Positive	1099	23.3	957	22.5	142	30.0	
Negative	3614	76.5	3291	77.4	323	68.3	
Unknown	13	0.3	5	0.1	8	1.7	
Cancer at surgical margin							0.30
Positive	274	5.8	252	5.9	22	4.7	
Negative	4452	94.2	4001	94.1	451	95.4	
NAC regimen							
Anthracycline/taxane					400	84.6	
Anthracycline					34	7.2	
Others					39	8.2	

*NAC* neoadjuvant chemotherapy, *SSM* skin-sparing mastectomy, *NSM* nipple-sparing mastectomy, *TE* tissue expander, *SBI* silicone breast implant, *SNB* sentinel node biopsy, *Ax* axillary dissection, *PMRT* post-mastectomy radiation therapy, *HER2* human epidermal growth factor receptor-2

## Safety

During a median follow-up period of 76.5 months (range 0–168), overall, postoperative complications were observed in 840 cases (17.8%). There were no significant differences in the incidence of complications between the NAC group (96 cases; 20.3%), and the control group (744 cases; 17.5%). We also assessed the correlations between postoperative complications and NAC regimens. Our results showed no significant correlations between them. Table 2 shows the associations between NAC and postoperative complications. The NAC group had significantly higher incidences of capsular contracture of the tissue expander (1.1%) and rupture (1.1%) of the SBI or TE than the control group, in which the incidences were 0.1% and 0.05%, respectively (*P* < 0.001, 0.001). In the NAC group, two of the four patients with capsular contracture had undergone PMRT, while the four with rupture had not. Table 3 shows the results of the regression analysis for postoperative complications, adjusted for factors such as BMI, smoking status, breast surgery type, reconstruction type, axillary surgery, NAC, PMRT and adjuvant chemotherapy. Overweight patients (OR 1.29; 95%CI 1.13–1.48) compared to those with normal BMI, those who underwent NSM/SSM (OR 1.60; 95%CI 1.45–1.76) compared to those who underwent total mastectomy, and those who received reconstruction with prosthesis (OR 1.28; 95%CI 1.15–1.43) compared to those who received reconstruction with autologous tissue, were found to have a significantly increased risk of developing postoperative complications. NAC was not, however, associated with an increased risk of postoperative complications.

**Table 2:** Complications

				Control			NAC			*P* value
Complications by procedure				Complications (N)	Overall (N)	%	Complications (N)	Overall (N)	%	
Mastectomy procedure		Reconstruction type								
Total Mastectomy		TE-SBI		175	1460	12.0	11	95	11.6	
		Direct SBI		0	2	0	0	2	0	
		Autologous		14	102	13.7	6	41	14.6	
SSM		TE-SBI		171	1254	13.6	13	98	13.3	
		Direct SBI		5	25	20.0	1	18	5.6	
		Autologous		58	169	34.3	8	29	27.6	
NSM		TE-SBI		253	997	25.4	37	133	27.8	
		Direct SBI		27	88	30.7	13	31	41.9	
		Autologous		41	156	26.3	7	26	26.9	
Overall				744	4248	17.5	96	473	20.3	0.13
Details of complications										
	Overall	Grade 2	Grade 3	Overall	Grade 2	Grade 3	Overall	Grade 2	Grade 3	
Hemorrhage	123 (2.6%)	85 (1.8)	38 (0.8)	101 (2.4%)	73 (1.7)	38 (0.9)	12 (2.5%)	12 (2.5)	0 (0)	0.83
Dehiscence	22 (0.4)	4 (0.08)	18 (0.4)	20 (0.5)	4 (0.09)	16(0.4)	2 (0.4)	0 (0)	2 (0.4)	0.89
Infection	175 (3.7)	45 (1.0)	130 (2.8)	152 (3.6)	38 (0.9)	114 (2.7)	23 (4.9)	7 (1.5)	16 (3.4)	0.16
Skin necrosis	277 (4.8)	152 (3.2)	75 (1.6)	208 (4.9)	141 (3.3)	67 (1.6)	19 (4.0)	11 (2.3)	8 (1.7)	0.4
Flap necrosis	12 (2.3)	3 (0.6)	9 (1.7)	8 (1.9)	1 (0.2)	7 (1.6)	4 (4.2)	2 (2.1)	2 (2.1)	0.18
Seroma	60 (1.3)	56 (1.2)	4 (0.08)	52 (1.2)	48 (1.1)	4 (0.09)	8 (1.7)	8 (1.7)	0 (0)	0.38
Marginal necrosis	135 (2.9)	95 (2.0)	40 (0.8)	117 (2.8)	82 (1.9)	35 (0.8)	18 (3.8)	13 (2.7)	5 (1.1)	0.19
Nipple necrosis	213 (14.9)	192 (13.4)	21 (1.5)	185 (14.9)	165 (13.3)	20 (1.6)	28 (14.7)	27 (14.2)	1 (0.5)	0.95
Capsular contracture	8 (0.2)	8 (0.2)	0 (0)	4 (0.1)	4 (0.1)	0 (0)	4 (1.1)	4 (1.1)	0 (0)	< 0.001
Malrotation	3 (0.07)			3 (0.08)			0 (0)			0.59
Rupture (TE/SBI)	6 (0.1) (2/4)			2 (0.05) (1/1)			4 (1.1) (1/3)			< 0.001
Prosthesis loss	119 (2.8)			103 (2.6)			16 (4.2)			0.07
Total flap loss	2 (0.3)			2 (0.5)			0 (0)			0.50
Details of complications by procedures					Grade 2	Grade 3		Grade 2	Grade 3	
Breast-surgery type	Reconstruction type									
Total mastectomy	TE-SBI									
Hemorrhage					15 (0.01)	7 (0.01)		1 (0.001)	0 (0)	
Dehiscence					1 (0.001)	5 (0.003)		0 (0)	1 (0.01)	
Infection					5 (0.003)	32 (0.02)		1 (0.01)	2 (0.02)	
Flap necrosis					45 (0.03)	32 (0.02)		1 (0.01)	1 (0.01)	
Seroma					3 (0.002)	1 (0.001)		2 (0.02)	0 (0)	
Marginal necrosis					24 (0.02)	15 (0.10)		2 (0.02)	0 (0)	
Capsular contracture					0 (0)	0 (0)		0 (0)	0 (0)	
Rupture						0 (0)			1 (0.01)	
Loss						26 (0.02)			3 (0.03)	
	Direct SBI									
None					0 (0)	0 (0)		0 (0)	0 (0)	
	Autologous									
Hemorrhage					0 (0)	1 (0.01)		1 (0.02)	0 (0)	
Dehiscence					0 (0)	0 (0)		0 (0)	0 (0)	
Infection					2 (0.02)	0 (0)		0 (0.0)	2 (0.05)	
Flap necrosis					4 (0.04)	1 (0.01)		1 (0.02)	2 (0.05)	
Seroma					4 (0.04)	0 (0)		0 (0)	0 (0)	
Marginal necrosis					2 (0.02)	0 (0)		0 (0)	0 (0)	
Flap loss						1 (0.01)			0 (0)	
SSM	TE-SBI									
Hemorrhage					21 (0.02)	8 (0.01)		2 (0.02)	0 (0)	
Dehiscence					0 (0)	0 (0)		0 (0)	0 (0)	
Infection					13 (0.01)	34 (0.03)		3 (0.03)	4 (0.04)	
Flap necrosis					44 (0.04)	20 (0.02)		2 (0.02)	1 (0.01)	
Seroma					8 (0.01)	2 (0.002)		1 (0.01)	0 (0)	
Marginal necrosis					21 (0.02)	7 (0.01)		1 (0.01)	0 (0)	
Capsular contracture					0 (0)	0 (0)		0 (0)	0 (0)	
Rupture						0 (0)			1 (0.01)	
Loss						32 (0.03)			3 (0.03)	
	Direct SBI									
Hemorrhage					0 (0)	0 (0)		0 (0)	0 (0)	
Dehiscence					0 (0)	0 (0)		0 (0)	0 (0)	
Infection					0 (0.0)	1 (0.04)		0 (0)	0 (0)	
Flap necrosis					0 (0)	0 (0)		0 (0)	1 (0.06)	
Seroma					0 (0)	0 (0)		0 (0)	0 (0)	
Marginal necrosis					2 (0.08)	1 (0.04)		0 (0)	0 (0)	
Capsular contracture					6 (0.24)	1 (0.04)		1 (0.06)	0 (0)	
Malrotation						1 (0.04)			0 (0)	
Loss						0 (0)			0 (0)	
	Autologous									
Hemorrhage					3 (0.02)	3 (0.02)		1 (0.03)	0 (0)	
Dehiscence					1 (0.01)	0 (0)		0 (0)	1 (0.03)	
Infection					5 (0.03)	0 (0)		0 (0)	0 (0)	
Flap necrosis					10 (0.06)	4 (0.02)		2 (0.07)	0 (0)	
Seroma					21 (0.12)	0 (0)		2 (0.07)	0 (0)	
Marginal necrosis					17 (0.10)	3 (0.02)		2 (0.07)	2 (0.07)	
Flap loss						1 (0.06)			0 (0)	
NSM	TE-SBI									
Hemorrhage					21 (0.02)	18 (0.02)		4 (0.03)	0 (0)	
Dehiscence					2 (0.002)	3 (0.003)		0 (0)	0 (0)	
Infection					10 (0.01)	46 (0.05)		3 (0.02)	6 (0.05)	
Flap necrosis					37 (0.04)	8 (0.01)		3 (0.02)	2 (0.02)	
Seroma					6 (0.01)	1 (0.001)		0 (0)	0 (0)	
Marginal necrosis					9 (0.01)	6 (0.01)		4 (0.03)	2 (0.02)	
Nipple necrosis					119 (0.12)	16 (0.02)		18 (0.14)	1 (0.01)	
Capsular contracture					2 (0.002)	2 (0.002)		3 (0.02)	0 (0)	
Malrotation						3 (0.003)			0 (0)	
Loss						40 (0.04)			6 (0.05)	
	Direct SBI									
Hemorrhage					1 (0.01)	1(0.01)		2 (0.06)	0 (0)	
Dehiscence					0 (0)	0 (0)		0 (0)	0 (0)	
Infection					1 (0.01)	1 (0.01)		0 (0)	2 (0.06)	
Flap necrosis					1 (0.01)	1 (0.01)		0 (0)	0 (0)	
Seroma					1 (0.01)	0 (0)		1 (0.03)	0 (0)	
Marginal necrosis					0 (0)	0 (0)		3 (0.10)	0 (0)	
Nipple necrosis					21 (0.24)	0 (0)		5 (0.16)	0 (0)	
Capsular contracture					2 (0.02)	0 (0)		0 (0)	0 (0)	
Rupture						1 (0.01)			2 (0.06)	
Loss						4 (0.05)			4 (0.13)	
	Autologous									
Hemorrhage					0 (0)	0 (0)		0 (0)	0 (0)	
Dehiscence					0 (0)	0 (0)		0 (0)	0 (0)	
Infection					2 (0.01)	0 (0)		0 (0)	0 (0)	
Flap necrosis					5 (0.03)	1 (0.01)		2 (0.08)	1 (0.04)	
Seroma					5 (0.03)	0 (0)		2 (0.08)	0 (0)	
Marginal necrosis					7 (0.05)	3 (0.02)		1 (0.04)	1 (0.04)	
Nipple necrosis					22 (0.14)	4 (0.03)		3 (0.12)	0 (0)	
Flap loss						0 (0)			0 (0)	

*NAC* neoadjuvant chemotherapy, *SSM* skin-sparing mastectomy, *NSM* nipple sparing mastectomy, *TE* tissue expander, *SBI* silicon breast implant

**Table 3:** Univariate and multivariate analyses for factors associated with postoperative complications

Variables	Category	Univariate		Multivariate	
		OR	95%CI	OR	95%CI
Body mass index	< 25	1		1	
	≥ 25	1.31	1.15–1.48	1.29	1.13–1.48
Smoking	No	1		1	
	Yes	1.02	0.95–1.11	0.98	0.90–1.06
Breast surgery type	Total mastectomy	1		1	
	SSM, NSM	1.63	1.49–1.79	1.60	1.45–1.76
Reconstruction type	Autologous	1		1	
	Prosthesis	1.36	1.22–1.51	1.28	1.15–1.43
Axillary surgery	None, SNB	1		1	
	Axillary dissection	1.24	1.05–1.47	1.07	0.89–1.30
Neoadjuvant chemotherapy	No	1		1	
	Yes	1.20	0.95–1.53	0.96	0.74–1.25
Post mastectomy radiation therapy	No	1		1	
	Yes	1.54	1.20–1.99	1.26	0.99–1.62
Adjuvant chemotherapy	No	1		1	
	Yes	1.8	1.02–1.37	1.08	0.91–1.29

*OR* odds ratio, *SSM* skin-sparing mastectomy, *NSM* nipple-sparing mastectomy, *SNB* sentinel node biopsy

## Oncologic outcome

**Fig. 1:**
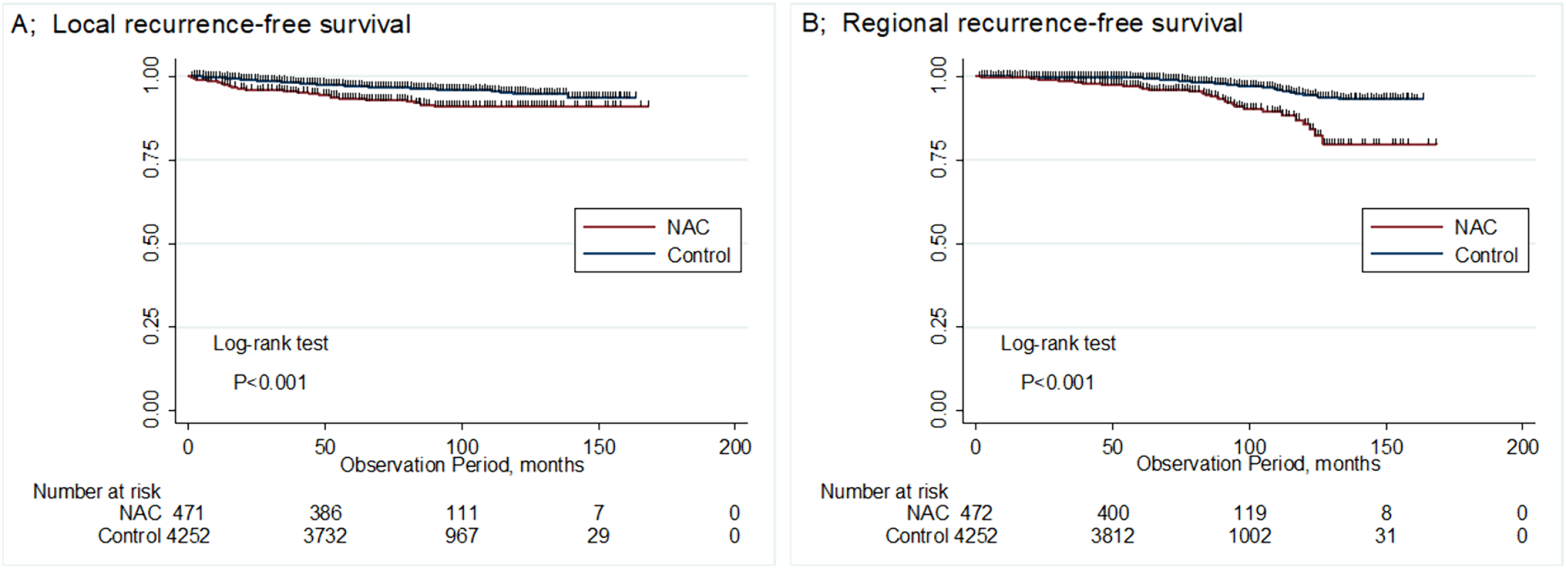
**a** Local recurrence-free survival curve; **b** regional recurrence-free survival curve

During a median follow-up period of 76.5 months (range 0–168), 36 patients (7.6%) in the NAC group and 147 (3.5%) in the control group developed local recurrence (*P* < 0.001). The 5-year LRFS was 93.1% (95% CI 0.90–0.95) in the NAC group and 97.1% (95% CI 0.97–0.98) in the control group. The rates of main local recurrence sites are as follows; subcutaneously fatty tissue, skin, nipple areola; 47.2%, 27.8%; 22.2% in the NAC group. Regional recurrence was detected in 35 patients (7.4%) in the NAC group and 85 (2.0%) in the control group (*P* < 0.001). The 5-year RRFS were 96.4% (95% CI 0.94–0.98) and 99.5% (95% CI 0.99–1.00). Kaplan–Meier survival curves for local recurrence (log-rank test, *P* < 0.001) and regional recurrence (log-rank test, *P* < 0.001) are shown in Fig. 1. Table 4 shows the results of univariate and multivariate analyses for LRFS and RRFS. Multivariate analysis for LRFS demonstrated pathological tumor size (HR 1.34; 95% CI 1.15–1.56), lymph node status (HR 0.70; 95% CI 0.52–0.95), lymphovascular invasion (HR 1.67; 95% CI 1.16–2.40), surgical technique (HR 1.81; 95% CI 1.49–2.22), PMRT (HR 0.18; 95% CI 0.07–0.44), and NAC (HR 2.00; 95% CI 1.33–3.04) to be factors significantly associated with local recurrence. After propensity score matching, however, there was no difference in local recurrence between the two groups. Multivariate analysis for RRFS demonstrated pathological tumor size (HR 1.31; 95% CI 1.08–1.59), lymphovascular invasion (HR 2.77; 95% CI 1.79–4.30), PMRT (HR 0.28; 95% CI 0.13–0.58), and NAC (HR 2.63; 95% CI 1.69–4.09) to be factors significantly associated with regional recurrence.

**Table 4:** Univariate and Multivariate analyses for factors associated with local and regional recurrences

Variables	Category	Local recurrence				Reginal recurrence								
		Univariate		Multivariate		Univariate		Multivariate						
		HR	95%CI	HR	95%CI	HR	95%CI	HR	95%CI					
pT	< 5 cm	1		1		1		1						
	≥ 5 cm	1.40	1.25–1.58	1.34	1.15–1.56	1.81	1.56–2.09	1.31	1.08–1.59					
pN	Negative	1		1		1		1						
	Positive	1.22	0.95–1.56	0.70	0.52–0.95	2.11	1.66–2.68	0.99	0.73–1.34					
Estrogen receptor	Negative	1		1		1		1						
	Positive	0.71	0.48–1.05	0.61	0.32–1.12	0.88	0.52–1.47	0.91	0.45–1.85					
Progesterone receptor	Negative	1		1		1		1						
	Positive	0.83	0.59–1.16	1.21	0.70–2.10	0.77	0.52–1.16	0.75	0.43–1.31					
HER2	Negative	1		1		1		1						
	Positive	1.01	0.67–1.52	0.89	0.57–1.39	0.87	0.52–1.45	0.66	0.36–1.18					
Lymphovascular invasion	Negative	1		1		1		1						
	Positive	1.97	1.46–2.67	1.67	1.16–2.40	4.11	2.87–5.90	2.77	1.79–4.30					
Surgery type	Bt	1		1		1		1						
	SSM/NSM	2.00	1.67–2.43	1.81	1.49–2.22	1.39	1.13–1.71	1.19	0.96–1.50					
PMRT	No	1		1		1		1						
	Yes	0.42	0.19–0.95	0.18	0.07–0.44	1.36	0.73–2.52	0.28	0.13–0.58					
Neoadjuvant chemotherapy	No	1		1		1		1						
	Yes	2.21	1.53–3.20	2.00	1.33–3.04	3.43	2.31–5.09	2.63	1.69–4.09					

*HER2* human epidermal growth factor-2, *Bt* total mastectomy, *SSM* skin-sparing mastectomy, *NSM* nipple-sparing mastectomy, *PMRT* post-mastectomy radiation therapy

## Discussion

This retrospective multicenter study showed that patients who received IBR after NAC did not have significantly higher rates of postoperative complications than those who did not receive NAC. To our knowledge, this is one of the largest studies to date evaluating the impact of NAC on the safety and long-term outcomes of patients undergoing IBR worldwide.

In this retrospective study, the postoperative complication rate was 20.3% in the NAC group. This result showed a favorable outcome compared to previous reports which indicated incidence of postoperative complications in patients who received NAC before IBR ranging from 23 to 39% [25–28]. In our study, the incidence of capsular contracture of the TE (1.1%) and ruptured prostheses (1.1%), including one TE and three SBI cases, were notably low in the NAC group; nonetheless, these rates were significantly higher than those in the control group. Capsular contracture of the SBI was not observed. However, it's important to note that the incidence of prosthesis loss (4.2%) and total flap loss (0%) was not significantly higher in the NAC group. These results also showed a favorable outcome compared to previous reports which indicated rates of prosthesis loss ranging from 8 to 26% and those of total flap loss ranging from 0 to 4% [26, 28]. We consider the following reasons for the rarity of capsular contracture of the SBI. In this study, we performed post-pectoral implant-based breast reconstruction using the textured surface implant, because the case collection period was before breast implant-associated anaplastic large cell lymphoma was reported, and the follow-up period (median; 76.5 months) may be inadequate.

Multivariate logistic regression analysis identified being over-weight, NSM/SSM, and reconstruction with TE/SBI as factors associated with an increased risk of complications. Our data and several meta-analyses focusing on IBR after NAC showed no significant increase in postoperative complications [21, 22].

The 5-year LRFS was 93.1% in the NAC group and 97.1% in the control group. The respective 5-year RRFS were 96.4% and 99.5%. The Cox regression model showed that the use of NAC was also a predictor of local and regional recurrences, along with large pathological tumor size, positive lymphovascular infiltration, NSM/SSM and no PMRT administration. The reasons for these observations could be attributed as follows; Firstly, patients who received NAC were more likely to have advanced stage breast cancer and to have hormone receptor negative and/or HER2 positive tumors with a high biological grade. However, following propensity score matching, the impact of NAC on local recurrence did not attain statistical significance. Secondary, patients receiving NAC more likely to undergo NSM, and in the NAC group, eight patients (4.2%) developed nipple recurrence, whereas this occurred in 11 patients (0.8%) in the control group. Thirdly, PMRT might be omitted in some cases for whom it was indicated due to fear of complications. To improve local control, PMRT was used for breast cancer patients with more than 4 positive nodes. The Japan Breast Cancer Society and National Comprehensive Cancer Network guidelines recommend using PMRT, regardless of the reconstruction approach [29, 30]. However, both patients and physicians might elect to forego PMRT if IBR has been undergone due to concerns regarding complications. In the American College of Surgeons Oncology Group Z1071 study, a prospective trial that evaluated the false negative rate of sentinel lymph node surgery after NAC among breast cancer patients with initial node positive disease, PMRT was significantly less common in patients undergoing IBR [31]. We need to provide meticulous follow-up for the patients who received NAC before IBR, particularly after undergoing NSM. However, several meta-analyses and recent reports have demonstrated that IBR after NAC had no impact on either overall or disease-free survival nor local recurrence [23–25]. Recently, Wu et al. examined oncological safety by applying the propensity score matching method to compare 323 patients who underwent IBR and 323 patients receiving conventional mastectomy alone after NAC. The median follow-up period in their study was 67 months. They experienced 3.7% of local recurrence, and 7.1% of regional recurrence in patients with IBR after NAC and found that there were no significant differences between the two groups in either local or regional recurrence, nor in rates of distant metastasis and overall survival [14]. In the past, a large proportion of patients receiving NAC undergo mastectomy as the surgical treatment either because breast- conserving surgery is not feasible or according to patient preference [32]. Although careful follow-up is required for post-operative local recurrence and adverse effects on TE and SBI, IBR now needs to be established as a standard procedure for patients treated with NAC to restore the physical image and positively influence the psychological well-being and quality of life of the patient.

The present study has several limitations. This was a retrospective study conducted at several institutions. Therapeutic strategies might have differed among them. However, the strengths of this study lie in the large number of subjects, the long period of follow-up and the detailed examination.

## Conclusion

The findings of this large retrospective study suggest that IBR after NAC is a safe procedure with an acceptable postoperative complication profile. IBR has become now well established as one of the standard procedures for patients treated with NAC, although careful follow-up is required regarding postoperative locoregional recurrence and adverse effects on TE and SBI.
